# Exploring the Human Health Impact of Artificial Turf Worldwide: A Systematic Review

**DOI:** 10.1177/11786302241306291

**Published:** 2024-12-17

**Authors:** Sebastian Ryan-Ndegwa, Reza Zamani, Tanimola Martins

**Affiliations:** Faculty of Health and Life Sciences, University of Exeter Medical School, University of Exeter, Exeter, UK

**Keywords:** Microplastics, artificial grass, human health, cancer risk, non-carcinogenic risk, polycyclic aromatic hydrocarbons, phthalates, heavy metals, toxicity

## Abstract

The growing use of artificial turf in place of natural turf in residential, recreational and commercial settings has raised concerns regarding its potential impact on human health. A systematic review of databases revealed 5673 articles of which, 30 were deemed eligible. Those performing total concentration analyses, bioaccessibility analyses or human health risk assessments (HHRAs) of artificial turf fibres or crumb rubber infill were of interest. Health hazards and risks were explored in relation to three groups of chemicals of concern: polycyclic aromatic hydrocarbons (PAH), heavy metals and other rubber additives. Twenty-five studies performed total concentration analyses on samples of artificial turf infill and/or turf fibres. Of these studies, median reported concentrations of eight PAHs, cadmium, mercury and zinc exceeded the European limits used. Eight studies performed bioaccessibility assays using synthetic biofluids and simulated organ systems. PAHs were not found to be bioaccessible except for benzo[a]pyrene in gastric fluid; heavy metals were bioaccessible except arsenic, and rubber additives were mostly bioaccessible except for three plasticisers: diisobutyl phthalate, benzyl butyl phthalate and dibutyl phthalate. Fourteen studies performed HHRAs to determine non-carcinogenic and carcinogenic risk. Cancer risks were identified for ingestion exposure to PAH in children with pica and heavy metal exposure via dermal, inhalation and ingestion pathways. Non-carcinogenic risks were identified for the ingestion of cobalt in a child spectator and the ingestion of arsenic, cobalt, thallium and zinc. Potentially hazardous concentrations of chemicals were found across both artificial turf infill and artificial turf fibre samples; bioaccessibility of these chemicals varied. Definitive conclusions were unable to be derived on the human health risks posed to users of artificial turf under real-world exposure scenarios. Future studies are recommended to explore the risks associated with the potential synergistic toxicities of chemical mixtures found in artificial turf.

## Background

**Figure 1.**
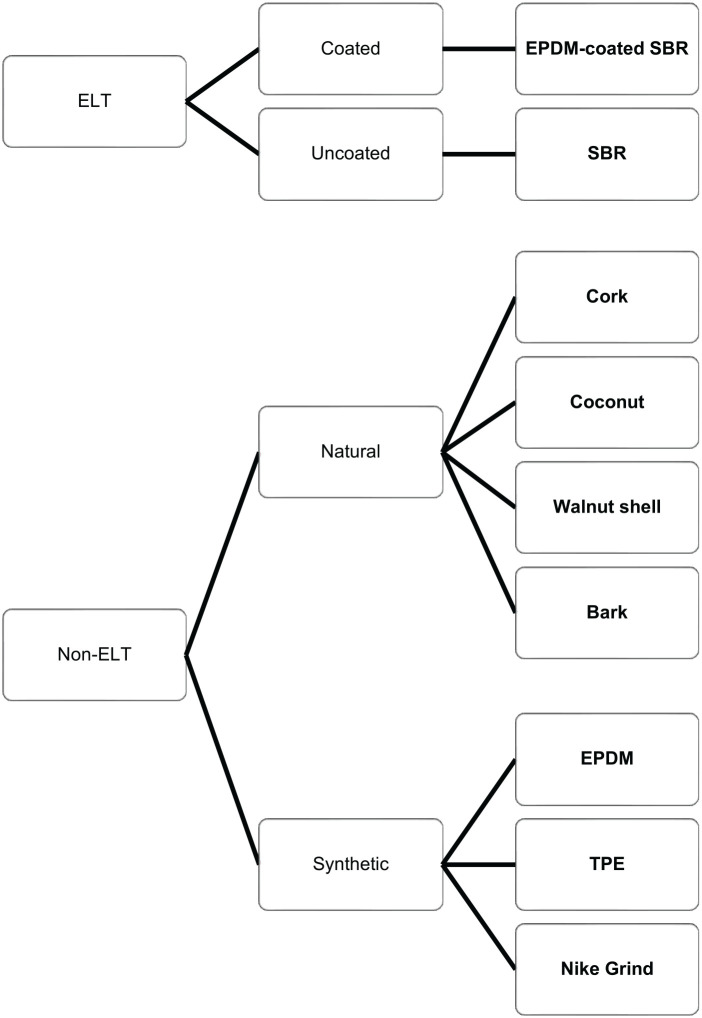
Types of artificial turf infill. Abbreviations: ELT, end-of-life tyre; EPDM, ethylene propylene diene monomer rubber; SBR, styrene butadiene rubber; TPE, thermoplastic elastomers.

There is a growing interest in the impact of microplastics on human health and modern life.^1-3^ A potentially overlooked contributor to microplastic pollution is artificial turf, which is increasingly used in recreational facilities and residential gardens.^4^ The most widely used artificial turf is third-generation (3G) artificial turf, which comprises grass-like polyethene fibres affixed to a rubber shock pad, with added infill to keep the fibres upright.^5^ This infill typically consists of a mixture of crumb rubber and sand. Crumb rubber infill is commonly made from granulated end-of-life tyres (ELT), consisting mostly of styrene-butadiene rubber (SBR).^6^ This ELT infill can either be used in its original state, or coated with ethylene-propylene diene monomer (EPDM) to add colour and potentially reduce environmental pollution. Other non-ELT types of infill are also used, either of synthetic or natural origin, as shown in Figure 1.

Microplastics are defined as solid plastic particles smaller than 5 mm in size;^7^ crumb rubber infill pieces are typically less than 1 mm in diameter, and a study found that 50% of artificial turf fibres sampled were less than 5 mm in size, classifying both as a sources of microplastics.^8^ Nanoplastics, defined as plastic particles in size ranging between 1 to 1000 nm,^9^ have also been detected in drainage water from artificial turf pitches.^10^ As part of wider bans on microplastics, the EU have placed a ban on the sale of granular synthetic polymer infill for use on synthetic sports surfaces, in effect from the 17th October 2031. This ban recognises the environmental impact of artificial turf; it has been suggested that artificial turf is responsible for 12% to 50% of global microplastic pollution.^11^ In Europe, approximately 16,000 tonnes of crumb rubber escapes artificial turf fields into the environment each year, primarily through rainfall runoff.^8^ An estimated 112 million square metres of artificial turf surfaces are currently installed in Europe alone,^12^ with the use of artificial turf becoming increasingly popular due to its versatility and low maintenance requirements.^4^ The proponents of artificial turf claim that by replacing natural turf with artificial turf on a single American football field, approximately 2 to 4 million litres of water could be saved annually.^13^ The Federation Internationale de Football Association (FIFA) also acknowledges the merits of artificial turf and has established the ‘FIFA Quality Program for Football Turf’ to provide quality marks to aid football clubs in choosing the best artificial turf solution for their needs.^14^

Indeed, artificial turf has been shown to have environmental merit over natural turf. A life cycle assessment showed that, over an entire life cycle, the global warming potential of artificial turf was less than half that of natural turf.^15^ Nevertheless, when the assessment was extended beyond global warming potential, artificial turf performed worse than natural turf in six separate categories of environmental impacts (including non-carcinogenic human toxicity and ecotoxicity of freshwater). This highlights the inter-locking issues of human and environmental health, ecotoxicity and sustainability which drive debate in discussions of artificial turf use, especially in comparison to the wide-ranging ecosystem services of natural turf.^16^ Anecdotal evidence suggesting a significant impact of artificial turf on human health has been suggested in the media.^11^ In 2019, a US soccer coach compiled a list of 260 young athletes over 5 years (mostly goalkeepers) diagnosed with lymphoma, who regularly played on artificial turf.^17^ Despite this, an ecological study investigating the incidence of malignant lymphoma in areas with a relatively high density of artificial turf found no correlation.^18^ A recent news report linked cases and deaths from glioblastoma, a rare brain cancer, to artificial turf use, sparking renewed interest in its chemical composition.^19^ The media representation of these health concerns has been described as controversial, potentially leading to ‘risk amplification’ without scientific basis, sparking debate regarding the current scientific consensus on the risk of crumb rubber in artificial turf.^20,21^ The presence of perfluoroalkyl and polyfluoroalkyl substances (PFASs), so-called ‘forever chemicals’,^22^ in artificial turf has received considerable media attention due to the links between PFAS exposure with liver and kidney disease, impaired immune function, reproductive toxicity and cancer.^23^ However, whilst research has shown that PFASs are present in artificial turf, PFASs in artificial turf are not prone to migrating from turf material to impact human health.^24^

Artificial turf samples contain a diverse range of chemicals, some of which have demonstrated in vitro bioactivities leading to effects such as endocrine, cardiometabolic and neurological toxicity.^25,26^ Notably, studies have identified polycyclic aromatic hydrocarbons (PAHs), heavy metals and other volatile organic compounds that are potentially hazardous to human health within artificial turf.^27^ Emerging studies have assessed how artificial turf pitches contain and release chemicals of concern (COCs) into the environment, with potential human exposure via inhalation of volatile substances released from artificial turf, accidental ingestion of crumb rubber infill, or dermal absorption, for example, through cuts and abrasions sustained during sporting activity. Depending on the bioaccessibility of these COCs via the route in which users are exposed, COCs have the potential to impact human health through increasing non-carcinogenic and carcinogenic risk. In addition, recent research has shown the presence of microplastics throughout the human body, including the brain,^28^ with research emerging on the potential health implications.^29^ Whilst artificial turf is a potential source of these microplastics, it remains to be determined what level of risk microplastics released from artificial turf pose to human health. The present review aims to describe the prevalence and characteristics of certain COCs present in artificial turf infill and fibres used worldwide, and to evaluate the human health risks posed to users of artificial turf.

## Methodology

**Figure 2.**
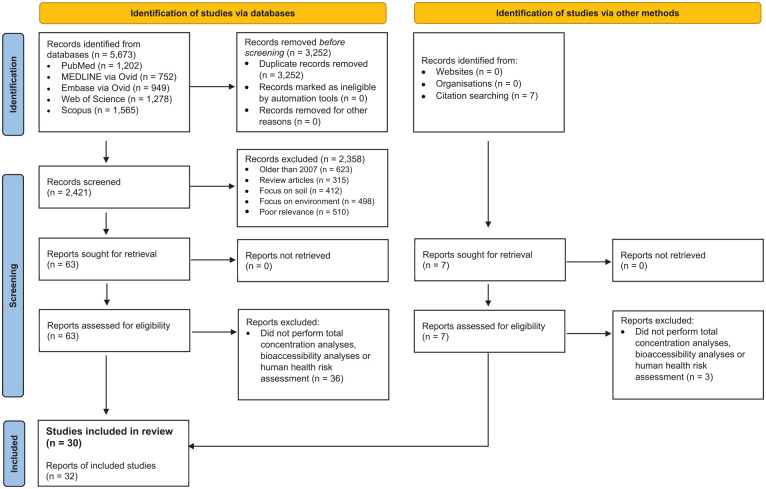
PRISMA flow diagram. Source: Adapted from Page et al.^46^ Abbreviation: PRISMA, Preferred Reporting Items for Systematic Reviews and Meta-Analyses.

On 29th June 2023, a systematic search was performed in five databases (MEDLINE, PubMed, Web of Science, Scopus and Embase) (see Figure 2). The search was constantly updated until the 3rd November 2023. The search strategy followed the Population, Interest and Context (PICo) framework. We defined ‘Population’ as people involved in activities on artificial turf-based surfaces, ‘Interest’ as health risks of artificial turf to humans, and ‘Context’ as the impact of exposure to artificial turf on human health. The following search query was used in all databases: (‘health’) AND (‘crumb rubber’ or ‘rubber crumb’ or ‘artificial turf*’ or ‘artificial grass*’ or ‘artificial pitch*’ or ‘synthetic turf*’ or ‘synthetic grass*’ or ‘synthetic pitch*’ or ‘playground*’). No publication date cut-off was used in the search. Grey literature articles were considered for triangulation purposes, however, were not included in the primary study selection.

### Eligibility criteria

Excluded studies focused on crumb rubber destined for other uses, for example, landscaping, mulch or road paving, did not provide evidence to assess the health impact of artificial turf or reported preliminary findings.

Eligible studies sampled crumb rubber infill or artificial turf fibre destined for artificial turf installation or already installed on artificial turf surfaces. They either provided evidence for evaluating or directly evaluated the health risks posed by artificial turf, that is, through providing total concentration values of COCs in artificial turf, through performing bioaccessibility analyses on COCs in artificial turf or by performing a human health risk assessment (HHRA).

Bioaccessibility analyses involve in vitro assays which determine the physiological solubility of a COC by determining the ratio between the total concentration of a COC found in an artificial turf sample and the concentration of that COC in the extraction media. The extraction media typically used are artificial sweat, saliva, and gastric or intestinal juice, which are tested either in isolation from one another or together, for example, in a simulated gastrointestinal tract.

HHRAs are a process used to determine the extent to which carcinogenic or non-carcinogenic health risks are posed to a population from exposure to substances, under certain circumstances. This multi-stage process considers many factors to determine health risk including the concentration at which COCs are hazardous, the bioaccessibility of COCs, the routes of exposure to COCs, the length of exposure to COCs and the level of exposure to COCs. Health risks can be determined for specific groups, for example, adult bystanders to a football match on artificial turf, through considering factors of exposure specific to certain users, for example, average inhalation rate.

Studies involving total concentration analyses and studies involving bioaccessibility analyses both provide evidence of potential health hazards. Through comparison of the results of total concentration analyses with published standards, total concentration analyses highlight where COCs are present at potentially hazardous concentrations within specific materials associated with artificial turf, that is, certain types of infill or fibres. Bioaccessibility analyses inform the extent to which COCs are absorbed by the body and by which organs, via which routes of exposure. HHRAs enable these hazards to be examined in the wider context of the environment in which humans are exposed to them, calculating the health risks posed. Consequently, results from total concentration and bioaccessibility analyses both input into HHRAs to estimate health risk; studies that performed any of these three types of analyses were therefore included.

### Study selection

The first stage of research selection included screening the title and abstract in accordance with the established PICo framework, and items were divided into accept, reject and maybe. Accept and maybe items were read in full for further assessment. Over 10% of initial search results were used in screen by SR-N and RZ to benchmark and reach convergence of selection criteria. One author, SR-N, then performed the search and the initial screening of records, with the second author, RZ, assisting in assessing full-text records for eligibility or all selected papers. Inconsistencies in study selection were discussed with the assistance of the third researcher, TM, until a final consensus was reached.

### Data extraction

We developed and piloted a data extraction spreadsheet using five randomly selected studies. Once finalised, SR-N extracted the data of eligible studies, which were then checked by RZ and TM. We extracted data on the study identifiers (author and country of sampling), sampling methodology and type of analysis performed, as shown in Supplementary Table 1. To summarise the wide range of COCs examined by the included studies, the results were categorised into three groups: PAHs, heavy metals and other rubber additives (constituting plasticisers, vulcanisers and antioxidants).

The reported PAHs were the eight PAHs identified by the European Chemicals Agency (ECHA) as being of greatest concern to human health through exposure to crumb rubber infill, due to evidence of their genotoxicity and mutagenicity.^30^ The reported PAHs were: benzo[a]pyrene (BaP), benzo[e]pyrene (BeP), benzo[a]anthracene (BaA), chrysene (CHR), benzo[b]fluoranthene (BbFA), benzo[j]fluoranthene (BjFA), benzo[k]fluoranthene (BkFA) and dibenzo[a,h]anthracene (DBAhA).

The five reported heavy metals reported (arsenic, lead, cadmium, chromium and mercury) were identified as being those most commonly associated with human toxicity.^31^ Zinc is a key component in the vulcanisation process of rubber and is consequently found in notably high concentrations in crumb rubber.^32^ Although zinc is not typically considered a toxic heavy metal,^33^ very high levels of zinc exposure may lead to toxicity in humans,^34^ therefore, it was reported in this review.

Nine rubber additives were reported in this review, as prioritised and reported in previously published health risk assessments on artificial turf.^6,35^ Additives from three main groups of chemicals used in the rubber manufacturing process were reported: plasticisers (which improve flexibility and mouldability),^36^ vulcanisers (which improve elasticity and strength)^37^ and antioxidants (which protect against environmental degradation).^38^ Four phthalate plasticisers were reported: di-ethylhexylphthalate (DEHP), dibutyl phthalate (DBP), diisobutyl phthalate (DIBP), and benzyl butyl phthalate (BBP), identified by the ECHA as being of greatest concern to human health due to their links to reproductive toxicity. Two phenolic vulcanisation agents were reported: 4-tert-octylphenol and bisphenol-A (BPA), both endocrine disruptors.^39,40^ Two thiazole vulcanisation accelerators were reported: benzothiazole, a respiratory irritant,^6^ and 2-mercaptobenzothiazole, an International Agency for Research on Cancer group 2A probable carcinogen linked to bladder cancer.^41^ One antioxidant, 6PPD, was reported as whilst it had not been included in previously published risk assessments, recent studies have shown that 6PPD-quinone, a 6PPD derivative produced with exposure to environmental ozone, may lead to adverse human health impacts including liver damage.^42^

Standards to evaluate the safety of total concentration levels of COCs within artificial turf were chosen based on their applicability to the reported results, that is, they related to human toxicology, total concentration analyses, were in appropriate units of measurement, and whether the documentation in which they were reported was accessible. Entry 50 of Annex XVII to EU Registration, Evaluation, Authorisation and Restriction of Chemicals (REACH) regulation (stating a summative limit of 20 mg/kg for eight PAHs)^30^ was chosen as an appropriate standard for PAHs. The ‘maximum concentration limits’ outlined by the Italian amateur football association Lega Nazionale Dilettanti (LND), derived from the German Deutsches Institut für Normung (DIN) V 18035-7 standard, were chosen as an appropriate standard for heavy metals.^43^ Entry 51 of Annex XVII to EU REACH regulation was chosen as an appropriate standard for the four phthalates reported in this paper (stating an individual limit of 1000 mg/kg for each phthalate).^44^ This particular standard was developed as a guideline to lower phthalate content in manufactured consumer goods, and potential exceedances do not directly relate to increased health hazards; it was chosen to provide an illustration of when a phthalate was present at a higher than typical concentration. No applicable standards were found for the other COCs included in this paper. Exceedances of these standards do not confer increased health risk; they were used to illustrate when a COC was present at a concentration deemed potentially hazardous to human health.

### Risk of bias assessment

We were unable to identify suitable risk of bias assessment tools to cover the range of methodologies used in the selected studies. Consequently, a novel risk of bias assessment tool was generated for the review based on the focused, extensive, applied and transparent (FEAT) principles.^45^ The tool assessed the risk of bias affecting the internal and external validity of each study, focusing on the sampling and analytical techniques implemented and the reported results. Four separate questions were devised for the tool, and each study was classified as ‘unmet’ or ‘met’, with unclassifiable studies rated as ‘not applicable’. Examples of reasons why the studies were classified as met or unmet were described in Table 1. RZ and SR-N rigorously processed and assessed study quality, based on the study characteristics described in Supplementary Table 1, and consequently evaluated the papers based on FEAT principles to assess the risk of bias. If studies could be classified as ‘met’ for 100% of the risk of bias questions, they were rated as ‘good’; if they ‘met’ between 50% and 100% of the questions, they were rated as ‘satisfactory’; if they met less than 50% of the questions, they were rated as ‘poor’. In addition to using a risk of bias tool, we checked whether the included studies provided information on the study funding sources and potential conflicts of interest between the authors or funding providers.

**Table 1.** Risk of bias assessment.

Main question	Was the sampling method appropriate for determining the composition of crumb rubber and/or turf fibre? (eg, in preventing contamination or decomposition of sample)	Was the sampling method appropriate for assessing human exposure to crumb rubber and/or turf fibre? (eg, player-worn direct vs pitch-located indirect air monitoring)	Were appropriate analytical techniques used in the characterisation of the crumb rubber and/or turf fibres?	Were the results reported appropriately? Are the findings generalisable to scenarios outside the experimental situation?	Rating
Sub-questions	1. Were visible contaminants removed from sample? 2. Were samples ever in contact with plastics? That is, stored in plastic containers or handled with plastic gloves 3. Were samples stored away from direct light? 4. Was the amount of sample collected reported? 5. Was sampling performed at multiple sites within each field?	1. Was sampling performed at body height? 2. Was sampling performed using multiple sampling devices? 3. Was a control sample collected off-pitch?	1. Did they use an experimental protocol that involved gas or ion chromatography-mass spectrometry, or inductively coupled plasma mass spectrometry for sample analysis?	1. Can this research be developed upon for future studies?	
Negev et al^59^	Not applicable	Not applicable	Met	Unmet	‘Satisfactory’
Mohamed et al^74^	Unmet	Unmet	Met	Met	‘Satisfactory’
Bocca et al^54^	Met	Not applicable	Met	Unmet	‘Satisfactory’
Celeiro et al^50^	Unmet	Not applicable	Met	Met	‘Satisfactory’
Menichini et al^47^	Met	Met	Met	Unmet	‘Satisfactory’
Pavilonis et al^67^	Unmet	Met	Met	Met	‘Satisfactory’
Ruffino et al^48^	Met	Unmet	Met	Met	‘Satisfactory’
Zhang et al^69^	Unmet	Met	Met	Met	‘Satisfactory’
Graça et al^76^	Unmet	Met	Met	Met	‘Satisfactory’
Pronk et al^51^	Unmet	Met	Met	Met	‘Satisfactory’
Marsili et al^53^	Unmet	Met	Met	Met	‘Satisfactory’
Simcox et al^71^	Met	Met	Met	Unmet	‘Satisfactory’
Zhang et al^58^	Not applicable	Not applicable	Met	Unmet	‘Satisfactory’
Zhang et al^65^	Not applicable	Not applicable	Met	Unmet	‘Satisfactory’
Peterson et al^68^	Not applicable	Not applicable	Not applicable	Met	‘Good’
Schneider et al^49,77,78^	Met	Met	Met	Met	‘Good’
Armada et al^75^	Met	Not applicable	Met	Met	‘Good’
Celeiro et al^56^	Met	Not applicable	Met	Met	‘Good’
Kim et al^60^	Not applicable	Met	Met	Met	‘Good’
Ginsberg et al^70^	Not applicable	Met	Met	Met	‘Good’
Grynkiewicz-Bylina et al^52^	Met	Not applicable	Met	Met	‘Good’
Schillirò et al^55^	Not applicable	Met	Met	Met	‘Good’
Nishi et al^61^	Not applicable	Met	Met	Met	‘Good’
Kawakami et al^62^	Not applicable	Met	Met	Met	‘Good’
Kubota et al^63^	Not applicable	Met	Met	Met	‘Good’
Kim et al^64^	Met	Met	Met	Met	‘Good’
Li et al^72^	Not applicable	Not applicable	Met	Met	‘Good’
Benoit and Demars^73^	Not applicable	Not applicable	Met	Met	‘Good’
Xie et al^66^	Not applicable	Met	Met	Met	‘Good’
Moreno et al^57^	Not applicable	Not applicable	Met	Met	‘Good’

If studies could be classified as ‘met’ for 100% of the risk of bias questions, they were rated as ‘good’; if they ‘met’ between 50% and 100% of the questions, they were rated as ‘satisfactory’; if they met less than 50% of the questions, they were rated as ‘poor’.

## Results

We retrieved 5673 records from the search. After removing duplicates and excluding studies based on eligibility criteria (see PRISMA diagram,^46^ Figure 2), 63 records were sought for full-text retrieval, 26 of which were included in the final analysis. A further search through the reference lists of eligible studies identified four additional studies. Overall, 30 studies were included in this systematic review.

### Study characteristics

The 30 eligible studies were published between 2008 and 2023. Ten of the included studies analysed samples from Europe,^47-57^ nine from Asia,^58-66^ seven from the United States of America,^67-73^ and one from Africa.^74^ Two studies analysed samples from multiple countries worldwide.^75,76^ Twenty-five studies analysed the total concentration of COCs in samples of crumb rubber and/or turf fibres used in artificial turf.^47-54,56,57,59-64,66,67,69,71-76^ HHRAs were conducted in 14 studies.^47,48,51,53,58,60,64,65,67,68,70,74,76,77^ Bioaccessibility analyses were conducted in eight studies.^60-64,67,69,78^ Ten studies analysed the air concentration of COCs associated with artificial turf,^47-51,53,55,71,73,74^ two of which performed headspace analysis on isolated samples of crumb rubber and/or turf fibres^50,73^ and eight of which performed on-pitch sampling. Analysis of leachate from artificial turf pitches was conducted in eight studies,^48,50-52,54,58,65,73^ five of which utilised laboratory-based analysis using isolated crumb rubber and/or turf fibre samples,^48,51,52,54,73^ three of which utilised on-pitch rainfall runoff sampling.^50,58,65^

### Risk of bias

Fourteen studies^47,48,50,51,53,54,58,59,65,67,69,71,74,76^ were classified as ‘satisfactory’, and 16 studies^49,52,55-57,60-64,66,68,70,72,73,75^ were classified as ‘good’. We reviewed the authors’ sources of funding and affiliations. Four studies^47,53,54,59^ did not report their funding sources or provide a conflict of interest statement. Nine studies^50,55,64,67,69-73^ provided an acknowledgement of their funding sources but did not provide a conflict of interest statement. Seventeen studies^48,49,51,52,56-58,60-63,65,66,68,74-76^ provided conflicts of interest statements with acknowledgements of funding sources. It was decided, however, that the absence of such a statement was not an admission of any conflict of interest (Table 1).

### Results for PAHs

Table 2 shows the median and maximum reported total concentrations of the eight PAHs per study from the analysis of crumb rubber and turf fibre samples. In summary, of the 15 studies that analysed the total concentration of the eight ECHA PAHs,^47,48,50-53,56,61,64,66,67,69,74,75,78^ three studies found median concentration values for the sum of all eight ECHA PAHs to be above the ECHA limit for TPE, ELT and unspecified crumb rubber (ie, crumb rubber of unspecified composition).^48,52,66^ Ten studies found the maximum concentration values for the sum of all eight ECHA PAHs to be above the ECHA limit for TPE, ELT, uncoated ELT, non-ELT and unspecified crumb rubber in addition to turf fibres.^47-49,52,53,56,66,69,74,75^ Xie et al^66^ reported the highest median concentration of 77.48 mg/kg and the highest maximum concentration of 496.25 mg/kg, both from crumb rubber samples.

**Table 2.** Total concentrations of polycyclic aromatic hydrocarbons (PAHs) reported in the included studies.

Authors	Material	benzo[a]pyrene		benzo[e]pyrene		benzo[a]anthracene		chrysene		benzo[b]fluoranthene		benzo[j]fluoranthene		benzo[k]fluoranthene		dibenzo[a,h]anthracene		SUM of 8 PAHs	
		Median	Max	Median	Max	Median	Max	Median	Max	Median	Max	Median	Max	Median	Max	Median	Max	Median	Max
Menichini et al^47^	TPE	0.05	0.05	/	/	0.15	0.29	0.72	1.43	0.02	0.03	see BbFA	see BbFA	/	/	/	/	1.82	1.80
	Coated ELT	1.18	2.30	/	/	0.10	0.15	0.98	0.99	0.26	0.46	see BbFA	see BbFA	/	/	0.03	0.03	2.54	3.93
	Uncoated ELT	2.21	10.70	/	/	0.35	0.43	1.16	2.38	0.42	1.78	see BbFA	see BbFA	/	/	/	/	8.25	30.58
Pavilonis et al^67^	Crumb rubber	/	/	/	/	/	/	/	/	/	/	/	/	/	/	/	/	/	/
	Turf fibre		/	/	/	/	/	/	/	/	/	/	/	/	/	/	/	/	/
Ruffino et al^48^	TPE	2.56	2.56	/	/	2.00	2.00	0.97	0.97	3.46	3.46	/	/	3.99	3.99	0.82	0.82	27.60	27.60
	ELT	1.26	1.41	/	/	10.38	15.30	2.37	4.21	4.37	8.81	/	/	3.73	5.02	2.52	8.13	24.62	42.88
Schneider et al^49^	Uncoated ELT	1.22	9.64	2.50	8.50	0.61	8.50	1.13	8.70	0.98	11.79	see BbFA	see BbFA	0.31	3.83	0.19	2.44	6.94	53.40
	Coated ELT	1.05	2.11	1.91	3.48	0.48	0.86	0.86	1.60	0.76	2.31	see BbFA	see BbFA	0.20	0.67	0.17	0.56	5.43	11.59
	Non-ELT	0.38	9.56	0.64	2.51	0.05	0.49	0.13	1.10	0.36	0.80	see BbFA	see BbFA	0.10	0.56	0.05	0.15	1.71	30.34
Armada et al^75^	Crumb rubber	0.51	15.00	1.80	18.00	0.30	17.00	1.33	29.00	0.51	17.00	see BbFA	see BbFA	0.12	8.00	0.28	3.00	4.85	107.00
Zhang et al^69^	Crumb rubber	0.61	8.58	/	/	0.65	1.26	1.65	7.55	0.83	3.39	/	/	0.18	7.29	0.62	/	4.54	31.59
	Turf fibre	0.08	0.08	/	/	/	/	/	/	/	/	/	/	/	/	/	/	0.08	0.08
Celeiro et al^56^	Crumb rubber	0.90	3.60	2.90	4.20	0.30	6.10	2.60	7.50	1.40	7.20	see BbFA	see BbFA	0.60	2.00	/	/	8.70	30.60
Pronk et al^51^	ELT	/	2.20	2.80	7.80	/	2.20	1.30	3.50	/	3.00	see BbFA	see BbFA	/	/	/	/	4.10	18.70
Grynkiewicz-Bylina et al^52^	ELT	3.02	9.24	4.90	12.29	3.23	4.85	8.68	21.10	4.84	6.34	1.08	1.71	1.82	2.36	0.84	21.03	28.41	78.92
	EPDM	/	/	/	/	/	/	15.30	15.30	/	/	/	/	/	/	/	/	15.30	15.30
Marsili et al^53^	ELT	0.24	0.66	/	/	0.25	1.61	0.92	3.42	3.23	15.72	/	/	0.56	3.62	0.27	0.57	10.94	25.60
	Non-ELT	0.17	0.17	/	/	0.27	0.27	0.70	0.70	1.56	1.56	/	/	0.35	0.35	0.43	0.43	3.48	3.48
Celeiro et al^50^	Crumb rubber	1.20	1.90	/	/	0.94	5.70	1.10	4.10	1.90	4.00	/	/	1.10	1.40	/	/	6.24	17.10
Nishi et al^61^	ELT	1.30	2.84	2.21	4.60	0.12	2.23	0.29	3.13	0.37	0.91	0.16	0.34	0.12	0.35	0.32	0.32	9.78	14.71
	EPDM	/	/	/	/	/	/	/	/	/	/	/	/	/	/	/	/	/	/
	TPE	/	/	/	/	/	/	0.23	0.23	/	/	/	/	/	/	/	/	0.23	0.23
Kim et al^64^ (MEAN)	Crumb rubber	/		/		/		/		/		/		/		/		/	
Xie et al^66^	Crumb rubber	1.05	1.26	/	/	8.23	28.93	0.79	1.74	1.89	2.90	/	/	1.74	2.11	/	/	1.82	28.93
	Turf fibre	2.53	7.75	/	/	183.83	348.05	5.76	39.60	115.35	496.25	/	/	32.43	155.90	/	/	77.48	496.25
Mohammed et al^74^ (MEAN)	Crumb rubber	113.40		/		286.70		330.90		450.90		/		159.30		97.50		450.90	

Abbreviations: BbFA, benzo[b]fluoranthene; ELT, end-of-life tire; EPDM, ethylene propylene diene monomer rubber; TPE, thermoplastic elastomer.

Total concentrations of eight PAHs in artificial turf infill and/or turf fibres reported by 15 studies that analysed PAHs. All reported values are in milligrams per kilogram. Underlined numbers denote where the reported concentration is above the limit of 20 mg/kg, as determined by the European Chemicals Agency (ECHA).^53^ ‘/’ denotes where studies failed to detect the chemical above the limit of detection, the chemical was not included as part of the analysis, or the value was not reported. Where studies have reported only mean values, as opposed to median or max, this has been denoted as ‘(MEAN)’ in the authors column.

### Results for heavy metals

Table 3 shows the median and maximum reported total concentrations of the five heavy metals per study from the analysis of crumb rubber and turf fibre samples. In summary, of the 19 studies that analysed the total concentration of heavy metals,^47-51,53,54,57,59,60,63,64,66,67,69,71,73,74,76^ in terms of median concentrations, cadmium and mercury exceeded the LND limit in whole artificial turf samples and non-ELT crumb rubber sampled in two studies.^53,59^ Zinc exceeded the LND limit in all reported studies (where tested), except Xie et al,^66^ in coated ELT, uncoated ELT, TPE, EPDM and unspecified crumb rubber.^47,48,50,53,54,57,63,69,73,76^ In terms of maximum concentrations, all five reported heavy metals exceeded the LND limit across all studies, with zinc exceeding the limit in all studies (where tested), in coated ELT, uncoated ELT, TPE, EPDM and unspecified crumb rubber; lead exceeding the limit in six studies, in whole artificial turf, ELT, unspecified crumb rubber and turf fibres;^48,59,67,71,74,76^ cadmium in five studies, in uncoated ELT, coated ELT, non-ELT crumb rubber and whole artificial turf;^49,51,53,59,76^ chromium in five studies, in whole artificial turf, turf fibres, EPDM, TPE, non-ELT and unspecified crumb rubber;^57,59,63,67,74^ mercury in one study, in whole artificial turf, and arsenic in one study, in whole artificial turf.^59^

**Table 3.** Total concentrations of heavy metals reported in the included studies.

Authors	Material	Arsenic		Lead		Cadmium		Chromium		Mercury		Zinc	
		Median	Max	Median	Max	Median	Max	Median	Max	Median	Max	Median	Max
Menichini et al^47^	TPE	0.54	0.94	44.50	46.00	0.24	0.37	52.50	56.00	0.05	0.05	3465.60	6813.00
	Coated ELT	0.18	0.24	28.00	28.00	1.01	1.90	4.00	6.20	0.08	0.08	10219.00	19375.00
	Uncoated ELT	0.12	0.41	22.00	26.00	1.40	1.90	1.20	4.60	0.07	0.16	13027.50	17772.00
Pavilonis et al^67^	Crumb rubber	/	0.80	/	17.00	/	1.10	/	16.00	/	/	/	/
	Turf fibre	/	4.00	/	4400.00	/	/	/	820.00	/	/	/	/
Ruffino et al^48^	TPE	/	/	34.30	34.30	/	/	/	/	/	/	5780.00	5780.00
	ELT	/	/	28.75	308.00	/	/	/	/	/	/	12500.00	15300.00
Schneider et al^49^	Uncoated ELT	/	/	23.00	85.00	1.80	48.90	/	/	/	/	/	/
	Coated ELT	/	/	26.50	37.00	1.80	4.00	/	/	/	/	/	/
	Non-ELT	/	/	5.50	33.00	0.30	0.50	/	/	/	/	/	/
Zhang et al^69^	Crumb rubber	0.28	3.55	5.20	53.50	0.22	0.41	0.90	1.68	/	/	7849.00	9988.00
	Turf fibre	/	/	2.80	2.80	/	/	3.93	3.93	/	/	/	/
Negev et al^59^	Artificial turf	5.00	480.00	7.50	2530.00	5.00	20.00	5.00	5710.00	5.00	100.00	/	/
Kim et al^50^	EPDM (<250 µm particles	/	/	2.00	7.00	/	/	/	/	/	/	/	/
	EPDM (>250 µm particles)	/	/	3.00	15.00	/	/	/	/	/	/	/	/
Graça et al^76^	Crumb rubber (indoor)	/	/	17.35	24.00	1.32	2.10	3.20	5.80	/	/	3944.00	4345.00
	Crumb rubber (outdoor)	1.40	2.00	16.00	214.00	0.80	4.00	4.15	35.00	/	/	4029.00	5246.00
Pronk et al^51^	ELT	/	/	/	35.00	/	2.10	/	/	/	/	/	17700.00
Marsili et al^53^	ELT	/	/	25.35	38.99	1.65	2.38	3.85	17.52	/	/	4530.00	13202.00
	Non-ELT	/	/	11.23	11.23	2.68	2.68	2.84	2.84	/	/	6462.00	6462.00
Bocca et al^54^	ELT	0.24	1.21	22.00	46.00	0.37	1.89	6.20	56.00	0.07	0.16	10229.00	19375.00
Celeiro et al^50^	Crumb rubber	0.48	0.71	23.70	23.90	0.84	1.11	1.35	1.50	/	/	14150.00	15100.00
Kubota et al^63^	ELT	0.58	0.85	21.80	29.20	0.88	1.65	3.08	20.00	0.01	0.02	20200.00	30800.00
	EPDM	1.41	2.08	12.20	19.70	0.23	0.23	23.10	22000.00	0.05	0.06	4140.00	4540.00
	TPE	5.32	8.57	1.20	1.57	0.42	0.42	44.60	138.00	0.002	0.004	453.00	1760.00
Simcox et al^71^	Turf fibre	/	/	60.15	76.50	/	/	/	/	/	/	/	/
	Crumb rubber	/	/	72.10	271.00	/	/	/	/	/	/	/	/
Kim et al^64^ (MEAN)	Crumb rubber	/		9.86		/		2.88		0.0002		2114.00	
Benoit and Demars^73^	Crumb rubber	/	/	22.05	33.10	0.84	1.39	/	/	/	/	19250.00	22200.00
Xie et al^66^	Turf fibre	/	/	0.12	0.27	/	/	0.01	0.02	/	/	0.28	0.35
	Crumb rubber	/	/	0.23	0.30	/	/	0.03	0.04	/	/	0.25	0.28
Mohammed et al^74^ (MEAN)	Crumb rubber	/		1156.00		/		145.00		/		1518.00	
Moreno et al^57^	ELT	1.05	1.10	21.15	21.20	0.55	0.60	83.45	157.30	/	/	17361.00	17622.00
LND limit^43^		20.00		100.00		2.00		150.00		1.00		150.00	

Abbreviations: ELT, end-of-life tire; EPDM, ethylene propylene diene monomer rubber; TPE, thermoplastic elastomer.

Total concentrations of six heavy metals in artificial turf infill and/or turf fibres reported by 19 studies that analysed metals. All reported values are in milligrams per kilogram. Underlined numbers denote where the reported concentration is above the LND limit.^43^ ‘/’ denotes where studies failed to detect the chemical above the limit of detection, the chemical was not included as part of the analysis, or the value was not reported. Where studies have reported only mean values, as opposed to median or max, this has been denoted as ‘(MEAN)’ in the authors column.

### Results for other rubber additives (plasticisers, vulcanisers and antioxidants)

Table 4 shows the median and maximum reported total concentrations of the nine additives per study from the analysis of crumb rubber and turf fibre samples. In summary, of the nine studies that analysed the total concentrations of the rubber additives,^49-51,56,59,62,64,72,75^ the maximum concentration of DBP exceeded the ECHA standard in one study, in unspecified crumb rubber, measuring 9470 mg/kg.^75^

**Table 4.** Total concentration of rubber additives reported in the included studies.

Authors	Material	DEHP		DBP		DIBP		BBP		4-tert-Octylphenol		Benzothiazole		2-mercaptobenzothiazole		6-PPD		BPA	
		Median	Max	Median	Max	Median	Max	Median	Max	Median	Max	Median	Max	Median	Max	Median	Max	Median	Max
Schneider et al^49^	Uncoated ELT	7.00	54.30	2.50	2.50	2.50	19.50	2.50	2.50	14.76	26.84	41.80	130.30	5.40	22.90	333.70	2064.80	2.77	10.24
	Coated ELT	7.80	21.70	2.50	2.50	2.50	17.40	2.50	2.50	4.63	22.75	6.80	85.00	4.10	13.80	36.50	170.50	2.08	9.56
	Non-ELT	4.40	11.20	2.50	5.40	2.50	5.30	2.50	6.70	0.50	5.50	2.50	81.50	2.50	563.30	54.30	2262.40	1.59	2.03
Armada et al^75^	Crumb rubber	28.00	9470.00	1.50	56.00	1.70	90.00	0.19	6.30	/	/	5.50	36.00	82.00	146.00	/	/	/	/
Negev et al^59^	Artificial turf	/	/	/	/	/	/	/	/	/	/	/	/	/	/	/	/	/	/
Celeiro et al^56^	Crumb rubber	8.00	28.00	0.70	3.90	1.00	3.80	0.30	1.70	/	/	2.70	34.00	39.00	62.00	/	/	/	/
Pronk et al^51^	ELT	7.60	27.20	/	/	/	2.30	/	/	4.80	22.40	2.70	6.30	2.60	7.60	/	/	0.50	2.50
Celeiro et al^50^	Crumb rubber	8.00	17.00	0.44	16.00	1.60	7.20	0.12	0.19	/	/	1.90	5.20	/	/	/	/	/	1.70
Kawakami et al^62^	ELT	11.00	67.00	/	/	/	/	/	/	24.00	41.00	109.00	151.00	30.00	114.00	1314.00	2916.00	/	/
	EPDM	/	/	/	/	/	/	/	/	/	/	25.00	92.00	514.00	1994.00	/	/	/	/
	TPE	/	/	/	/	/	/	/	/	/	/	/	/	1.70	2.30	/	/	/	/
Kim et al^64^ (MEAN)	Crumb rubber	0.41		/		/		/		/		/		/		/		/	
Li et al^72^	ELT	/	/	/	/	/	/	/	/	48.00	48.00	48.00	48.00	/	/	/	/	/	/
	Non-ELT	/	/	/	/	/	/	/	/	5.40	5.40	10.00	10.00	/	/	/	/	/	/
ECHA limit^44^		1000		1000		1000		1000		*		*		*		*		*	

Abbreviations: ELT, end-of-life tire; EPDM, ethylene propylene diene monomer rubber; TPE, thermoplastic elastomer.

Total concentration of nine rubber additives in artificial turf infill and/or turf fibres reported by nine studies that analysed rubber additives. All reported values are in milligrams per kilogram. Underlined numbers denote where the reported concentration is above the limit, as determined by the ECHA.^44^ ‘/’ denotes where studies failed to detect the chemical above the limit of detection, the chemical was not included as part of the analysis, or the value was not reported. Where studies have reported only mean values, as opposed to median or max, this has been denoted as ‘(MEAN)’ in the authors column. ‘*’ denotes chemicals for which published safety limits could not be identified.

### Bioaccessibility analyses of COCs

Table 5 shows the bioaccessibility results from the eight included studies that performed bioaccessibility assays.^60-64,67,69,78^

**Table 5.** Results of bioaccessibility assays reported in the included studies.

Study	PAH bioaccessibility	Heavy metal bioaccessibility	Other rubber additive bioaccessibility
Nishi et al^61^	• None of the ECHA PAHs were found to be above the LOQ in artificial gastric juice and intestinal juice	n/a	n/a
Schneider et al^78^	• None of the ECHA PAHs were found to be above the LOQ in artificial saliva or sweat.	n/a	• 4-tert-octylphenol was extracted in synthetic saliva, gastric juice and sweat. • Benzothiazole was extracted in synthetic sweat. • One of the four ECHA REACH phthalates, DEHP, was extracted in synthetic sweat. • BPA was extracted in synthetic sweat.
Pavilonis et al^67^	• Six out of the eight PAHs listed by the ECHA REACH were measured; none were found to be above the LOD in both synthetic biofluid extraction and total extraction methods.	• Arsenic was not detected above LOQs in any synthetic biofluid. • Lead was detected at greater concentrations in synthetic gastric fluid than in other synthetic biofluids. • Cadmium was detected in synthetic sweat. • Higher yields of lead and cadmium were extracted by synthetic digestive biofluids than by acid extraction methods in some samples.	• 4-tert-octylphenol was extracted in synthetic saliva, sweat and lung fluid. • Ten times more 4-tert-octylphenol was extracted in synthetic sweat than the concentration detected from total extraction. • Benzothiazole was not extracted in any synthetic biofluids; a dimer of benzothiazole, 2.2 benzothiazole, was extracted in synthetic digestive fluid.
Zhang et al^69^	• A 2.95% fraction of BaP was bioaccessible in synthetic gastric fluid.	• Arsenic was not detected above LOQs in any synthetic biofluid. • Lead was detected at greater concentrations in synthetic gastric fluid than in other synthetic biofluids • Lead was detected from a turf fibre sample in synthetic intestinal fluid, but not from a crumb rubber sample • Chromium was detected in synthetic gastric juice and saliva	n/a
Kim et al^60^	n/a	• Arsenic was not detected above LOQs in any synthetic biofluid. • Lead had a greater bioaccessibility in crumb rubber with a particle size smaller than 250 µm in an artificial gastric model (synthetic digestive juice, duodenal juice and bile juice) than in crumb with a particle size larger than 250 µm.	n/a
Kubota et al^63^	n/a	• Arsenic was not detected above LOQs in any synthetic biofluid. • Lead was extracted in synthetic gastric juice, saliva and sweat with low elution rates • Cadmium was not detected above LOQs in any synthetic biofluid • Chromium was extracted in synthetic gastric juice with low elution rates • Zinc was extracted in synthetic gastric juice, saliva and sweat with high elution rates in gastric juice	n/a
Kim et al^64^	n/a	• Arsenic was not detected above LOQs in any synthetic biofluid. • The bioaccessibility of lead from turf fibre samples was greater than from crumb rubber samples • Cadmium was detected in synthetic gastric fluid • Chromium was detected in synthetic gastric fluid.	n/a
Kawakami et al^62^	n/a	n/a	• 4-tert-octylphenol was extracted in gastric juice in one sample with a low elution rate • Benzothiazole was extracted in synthetic gastric juice, intestinal juice and saliva and sweat with high elution rates, especially in gastric juice • 2-mercaptobenzothiazole was extracted in synthetic gastric juice, intestinal juice and saliva with low elution rates • DEHP was not detected above LOQs in any synthetic biofluid • 6PPD was extracted in synthetic gastric juice, intestinal juice and saliva with low elution rates

Abbreviations: BPA, bisphenol-A; DEHP, di-ethylhexylphthalate; ECHA, European Chemicals Agency; LOD, limit of detection; LOQ, limit of quantification; PAH, polycyclic aromatic hydrocarbons; REACH, Registration, Evaluation, Authorisation and Restriction of Chemicals.

Reported findings from the eight included studies that performed bioaccessibility assays on polyaromatic hydrocarbons, heavy metals and other rubber additives.

### Human health risk assessments

Table 6 shows the HHRA results from the 14 included studies that performed HHRAs.^47,48,51,53,58,60,64,65,67,68,70,74,76,77^

**Table 6.** Results of human health risk assessments reported in the included studies.

Authors	Carcinogenic risk	Non-carcinogenic risk
Menichini et al^47^	• A preliminary risk assessment for inhalation exposure to BaP was performed, calculating a mean annual lifetime exposure of 0.025 ng/m^3^ BaP which is 2.5% of the EU Directive 2004/107/EC limit of 1 ng/m^3^ in ambient air.	n/a
Peterson et al^68^	• ELCR values calculated were all below the *de minimis* US EPA limit of 1 × 10^−6^ µg/m^3^, with the highest ELCR also associated with the child spectator (9 × 10^−7^). • Arsenic, PAHs and other organic compounds were the greatest overall contributors to cancer risk. • Cancer risks were greater in natural soil fields than in artificial turf fields for the composite player (indoor and outdoor), outdoor player and child spectator, all of which were above the *de minimis* EPA limit but below the EPA acceptable risk threshold of 1 × 10^−4^. • The cancer risk was lower in natural soil fields than in artificial turf fields for the adult spectator.	• Noncancer TOSHIs were calculated for a range of exposure subjects. • The highest TOSHI value (0.96) was for the endocrine system in a child spectator, with the overall HI for a child spectator being equal to 1. • The majority of noncancer impact for the child spectator resulted from estimated cobalt ingestion and overall, thallium, cobalt and 4-tert-octylphenol were the largest contributors to noncancer hazard.
Zhang et al^58^	• Explored the health risk of metals present in rainfall runoff from artificial turf pitches, focusing on dermal exposure. • Calculated that cancer risks were above the EPA acceptable risk threshold for chromium exposure (1.82 × 10^−4^) and arsenic exposure (1.23 × 10^−4^), both indicating an increased risk of cancer for pitch users.	• HQs calculated to be well below 1 for zinc, lead, copper, and manganese exposure from rainfall runoff.
Zhang et al^65^	• Explored the health risk of metals present in rainfall runoff from artificial turf pitches, focusing on dermal exposure. • After the first rainfall event, the ELCR calculated was below the EPA *de minimis* level. After the second and third consecutive rainfall events, the ELCR was calculated to be above the EPA *de minimis* level, but below the EPA acceptable risk threshold.	n/a
Ruffino et al^48^	• Through exploring the dermal route of exposure via direct dermal contact and contact with rainwater runoff, cancer risk was greater in an adult player than a child player. The ELCR was below the EPA *de minimis* limit.	• In contrast to their findings for carcinogenic risk, noncarcinogenic risk was greater for a child player than for an adult player. • The greatest contributors to noncarcinogenic risk in the child player were benzo[g,h,i]perylene, zinc and benzene from dermal contact with rainwater. • The cumulative noncarcinogenic risk was below 1 for all exposure scenarios on all fields. • Compared with synthetic rubber (styrene-butadiene) infill fields, fields with TPE infill had the lowest noncarcinogenic risk.
Schneider et al^77^	• The ELCR for dermal and oral exposure to six of the eight ECHA PAHs was below the EPA *de minimis* limit.	• RCRs for non-carcinogenic risk were all below 1 • Risks were greater via oral exposure than via dermal exposure. • BPA, 6PPD and aniline were the largest contributors to non-carcinogenic risk in both goalkeepers and players, aged 7 to 50 years. • Risks were consistent across players and goalkeepers, but were highest in adults (18-35 years), compared to other age groups.
Ginsberg et al^70^	• Calculated cancer risks which were up to threefold greater in indoor artificial turf pitches than in outdoor pitches, with cumulative cancer risks for children and adults indoors equal to the EPA *de minimis* level of 1 × 10^−6^.	• Calculated HQ for acute exposure which was greater than for chronic exposure, driven by the acute potential for respiratory irritation caused by benzothiazole; however, overall, the HQ did not exceed 1.
Graça et al^76^	• Explored the health risk of metal exposure via ingestion and dermal pathways, across five exposure groups (young/adult athlete, coach, child/adult bystander). • The adult bystander group was not applicable for the ingestion pathway. • For the ingestion pathway, cancer risks were above the EPA *de minimis* level but below the EPA acceptable risk threshold for As (all exposure groups) and in the young athlete group for chromium and lead. • Chromium was reported to be the largest contributor to cancer risk as As was found at a sample concentration below 10% (7%). • For the dermal pathway, all cancer risks were below the EPA *de minimis* level.	• For the ingestion pathway, calculated HQs were above 1 in arsenic, cobalt, thallium and zinc, with the HI being highest in the child bystander exposure group. • Thallium had the highest HQ (67.22 in child bystander); however, it was detected in only 2% of the samples. • Regarding the metals detected in more than 10% of the samples, zinc was the greatest contributor to the HI for the ingestion pathway (HQ of 6.71 in child bystander). • No HQs above 1 were found for the dermal pathway in any exposure group.
Pronk et al^51^	• In terms of PAH-associated cancer risk, ELCRs were at the EPA *de minimis* level for field players and above the EPA *de minimis* level for goalkeepers; however, they were below the acceptable risk threshold for the oral route of exposure to the eight ECHA PAHs. • Under the worst-case scenario (100% bioaccessibility, day exposure), exposure levels for cadmium and cobalt were below Dutch safety limits,^79^ whereas lead was above Dutch safety limits for dermal and oral routes of exposure in a 4-year-old child and 7-year-old goalkeeper. • After calculating lifetime exposure, however, all routes of lead exposure were below the Dutch safety limits for all users.	• For non-carcinogenic risk associated with benzothiazoles and phthalates, calculated HI was well below 1. • The HI for BPA-associated non-carcinogenic risk was equal to 1 for a worst-case scenario exposure for a 7-year-old goalkeeper for the dermal route of exposure, however, when lifetime exposure is calculated the HI is well below 1.
Marsili et al^53^	• Explored the health risk of six PAHs separately across nine artificial turf samples. • The ELCR values were above the EPA *de minimis* level but below the acceptable risk threshold. • In addition, the toxicity equivalent was calculated to determine the average daily exposure to PAHs for a 70 kg athlete. • A chronic exposure of 0.06 to 0.44 µg/kg bw per day was calculated for BaP equivalents, which is well above the virtually safe dose as determined by the EC Scientific Committee on Food (which implies an ELCR lower than the EPA *de minimis* level of 1 × 10^−6^) of 0.00057-0.005 µg/kg bw per day in food.	• Calculated HI less than 1.
Kim et al^64^	• Explored inhalation, ingestion and dermal exposure to artificial turf pitches installed in school playgrounds and athletic facilities. • Carcinogenic risk was assessed based on benzene and eight unspecified PAHs. • The highest ELCR was in children with pica, an eating disorder involving the consumption of inedible material, such as crumb rubber. • In the worst exposure scenario, the ELCR for children with pica was above the EPA *de minimis* level and at the acceptable risk threshold. • In an average exposure scenario, adults (50 years) had the highest ELCR which was at the EPA *de minimis* level.	• The non-carcinogenic risk assessment was based on xylene, toluene, ethylbenzene, formaldehyde, five heavy metals (cadmium, chromium, mercury, lead and zinc) and four unspecified PAHs. • In an average exposure scenario, the HI for all users were at or below 0.1, except in children with pica for which the HI was above 0.1. • In the worst exposure scenario, a HI above 1 was reported in children with pica.
Mohamed et al^74^	• Explored the health risk of heavy metal and PAH exposure via ingestion, inhalation and dermal pathways across different age groups. • All ages from 3 to 70 years had total cancer risks above the EPA *de minimis* level, with those aged 3 to 6 years and those aged 7 to 15 years having total cancer risks above the EPA acceptable risk threshold. • When the total cancer risk of only heavy metals is considered, all ages 3 to 70 have total cancer risk above the acceptable risk threshold. • Among the heavy metals tested, those contributing the most to cancer risk from the highest impact to the lowest were chromium, lead, cobalt and nickel. • Among the PAHs tested, those contributing the most to cancer risk from highest impact to lowest were: benzo(a)pyrene, dibenzo(a,h)anthracene, benzo(b)fluoranthene and benzo(a)fluoranthene.	• Calculated HI less than 1.
Pavilonis et al^67^	n/a	• Explored the non-carcinogenic health risk of five metals (arsenic, cadmium, chromium, copper and vanadium). • Across all sampled age ranges, (ages 6-19+ years), dermal exposure was found to be the primary route of exposure and inhalation exposure was found to be ‘inconsequential’. • No HI for any metal exposure in any age group was above 1, which would have represented a potential for an adverse health effect to occur.
Kim et al^60^	n/a	• When comparing the HQ of lead ingestion in school-age children (7-18 years) using EPDM infill artificial turf, the highest hazard level reported was in younger elementary school students (7-9 years), followed by older elementary students (10-12 years) and finally, middle and high school students (13-18 years). • The HQ was two times greater for ingestion of EPDM infill particles smaller than 250 µm than for the ingestion of particles larger than 250 µm. • Korean Environmental Health Law considers elementary school-age students as a sensitive group; therefore, 0.1 instead of 1 is suggested as the limit above which adverse health effects may occur. • In elementary school students who played on artificial turf with EPDM infill particles smaller than 250 µm, the mean HQ was above 0.1 in normal and extreme exposure scenarios, but when infill particles larger than 250 µm were used, the mean HQ was only above 0.1 in extreme exposure scenarios.

Abbreviations: BaP, benzo[a]pyrene; BPA, bisphenol-A; ECHA, European Chemicals Agency; ELCR, excess lifetime cancer risk; EPA, US Environmental Protection Agency; EPDM, ethylene propylene diene monomer; HI, hazard index; HQ, hazard quotient; PAH, polycyclic aromatic hydrocarbon; RCR, risk characterisation ratios.; TOSHI, target organic specific hazard index; TPE, thermoplastic elastomer.

Reported findings on cancer and noncancer risk from 15 included studies that performed human health risk assessments.

In describing cancer risk, excess lifetime cancer risk (ELCR) refers to the additional risk of developing cancer over the course of a subject’s lifetime, from exposure to cancer-causing chemicals. The US Environmental Protection Agency (US EPA) has an established scale of cancer risk^80^ from its ‘de minimis’ level at which a cancer risk becomes a concern (1 × 10^−6^) to the acceptable risk threshold (1 × 10^−4^), beyond which is considered highly concerning.

In describing non-carcinogenic risk, the hazard quotient (HQ) refers to the ratio between the concentration of a single chemical to which the subject is exposed and its maximum recorded safe concentration. The hazard index refers to the sum of HQs calculated for a mixture of chemicals. For both HQs and HIs, a score equal to or less than 1 represents no likelihood of adverse non-carcinogenic health effects occurring. A score above 1 indicates the potential for adverse non-carcinogenic health effects to occur.

## Discussion

### General interpretation of results

In this paper, we reviewed studies in which crumb rubber and turf fibres were sampled from artificial turf in 101 major locations (countries or states) at 419 sampling sites, with 3209 individual samples taken. These sampling sites primarily consisted of indoor and outdoor sports pitches as well as playgrounds. The sampling and analysis methods used were equally wide ranging, with studies investigating the impact of atmospheric temperature on COC volatility, the effect of crumb rubber particle size on COC toxicity and the mutagenicity of crumb rubber in vitro.

The main source of PAHs in crumb rubber is from the use of extender oils and carbon black in the manufacturing process of rubber.^81^ EPDM and TPE crumb rubbers are typically manufactured using paraffinic extender oils, which contain lower levels of PAH compared to the aromatic extender oils used in SBR tyre manufacturing, eventually becoming ELT crumb rubber.^6^ Despite this, of the three studies in which the median PAH concentrations exceeded the ECHA limit, Ruffino et al^48^ reported that the PAH concentration of TPE infill exceeded the ECHA limit, alongside ELT infill. Xie et al^66^ notably reported a maximum cumulative PAH concentration of 496 mg/kg in a turf fibre sample, nearly 25 times above the ECHA limit.^30^ The high level of PAHs in turf fibre was likely due to the high sorption of PAHs to polyethene, which acts as a sink for PAHs present either as manufacturing contaminants or environmental contaminants.^82^ Pavilonis et al^67^ and Kim et al^64^ were unable to detect any PAHs above the limit of detection (LOD) in their crumb rubber sample. Whilst there is no standardised method for the quantification of PAHs in plastics or rubber materials, both studies clearly reported their quantification methodology for PAHs, which was consistent with other reported studies’ methodologies.^81^

Median concentrations of cadmium and mercury exceeding the LND limit were reported by Negev et al^59^ (whole artificial turf sample) and Marsili et al^53^ (non-ELT infill). Mercury may be present due to contamination from environmental pollution, for example, nearby coal combustion.^83^ Cadmium may be present in crumb rubber as an attendant product of zinc oxide (ZnO),^84^ which is present in high concentrations in rubber, due to its use as a vulcanisation accelerator.^32^ This use of ZnO in rubber manufacturing also provides evidence to explain why the median concentrations of zinc exceeded the LND limit in all reported studies (where reported), except Xie et al.^66^ This is further supported by the lack of zinc detection in the reported turf fibre samples, except for the low concentration reported by Xie et al.^66^ For the maximum reported values, exceedances of the LND limits were found across all the heavy metals reported. The largest exceedances were reported by: Pavilonis et al,^67^ who reported a maximum lead concentration of 4400 mg/kg in a turf fibre sample (LND limit = 100 mg/kg); Negev et al,^59^ who reported a maximum arsenic concentration of 480 mg/kg (LND limit = 20 mg/kg), and a maximum mercury concentration of 100 mg/kg (LND limit = 1 mg/kg) in a whole artificial turf sample; Schneider et al,^49^ who reported a maximum cadmium concentration of 49 mg/kg in an uncoated ELT infill sample (LND limit = 2 mg/kg) and Kubota et al,^63^ who reported a maximum chromium concentration of 22,000 mg/kg (LND limit = 150 mg/kg) in an EPDM infill sample and a maximum zinc concentration of 30,800 mg/kg in an ELT infill sample. Despite some current restrictions,^85^ lead chromate has been used as a yellow pigment in plastics such as turf fibre and rubbers;^86,87^ the high reported concentration of lead in turf fibre and chromium in EPDM (which may have been coloured crumb), may result from the use of this pigment. It is important to note that whilst the concentration of zinc across reported studies was very high, zinc toxicity is rare,^34^ and zinc oxide has widespread use in cosmetics, that is, as a UV filter in sunscreens.^88^ However, efforts are underway to find alternative vulcanising agents to ZnO, as zinc is a significant environmental pollutant, and is associated with aquatic toxicity.^89^

As appropriate standards were only identified for the four ECHA phthalates (1000 mg/kg),^44^ the only reported exceedance was by an unspecified crumb rubber sample analysed by Armada et al,^75^ who reported a maximum DEHP concentration of 9900 mg/kg. However, the median value for DEHP reported by Armada et al^75^ was 28 mg/kg, suggesting that the maximum value may have represented an extreme outlier.

Despite some COCs being reported at concerning concentrations in samples of crumb rubber and turf fibre, it is important to consider the bioaccessibility of these chemicals. None of the eight ECHA PAHs were found to be bioaccessible in saliva, sweat or intestinal fluid except for BaP,^61,67,69,78^ the most carcinogenic PAH, which was bioaccessible in gastric fluid.^69^ For heavy metals, no bioaccessibility data were reported for mercury and zinc, but all other reported heavy metals, except arsenic, were bioaccessible. For rubber additives, all reported COCs except three of the four ECHA phthalates (DIBP, BBP and DBP) were bioaccessible. Consequently, exceedances of the limits discussed did not necessarily equate to increased hazards to human health. Nevertheless, due to the methodology by which the limits discussed are set, whilst the concentration levels of COCs which do not exceed their limit may be considered ‘acceptable’, this does not claim the concentration of those COCs to be necessarily ‘safe’. By utilising a wider gamut of variables, HHRAs represent a model to help elucidate the health risks posed by exposure to these COCs.

Many studies used an HHRA model directly taken or adapted from the US EPA’s list of appropriate models that can determine estimates of cancer and noncancer risk. Each model follows a four-step process:^90^ (1) identify the health problems caused by COCs, (2) quantify the concentration and duration of exposure, (3) consider the different health problems that occur at different levels of exposure, and (4) calculate the extra risk of health problems occurring from COC exposure.

Studies have reached mixed conclusions regarding the severity of cancer risk. Exceedances of the EPA upper acceptable risk threshold were reported for dermal chromium and arsenic exposure from rainfall runoff,^58^ ingestion exposure to PAHs in children with pica^64^ and heavy metal exposure via dermal, inhalation and ingestion in artificial turf users aged 3 to 70 years.^74^

Cancer risks above or at the EPA *de minimis* level but below the acceptable risk threshold were reported for inhalation exposure to PAHs in indoor artificial turf pitches,^70^ ingestion exposure to arsenic in adults and children, ingestion exposure to chromium and lead in young athletes,^76^ ingestion exposure to the eight ECHA PAHs in artificial turf field players and goalkeepers^51^ and PAH exposure in 50-year-old adults. Peterson et al^68^ found a greater cancer risk for natural soil than for artificial turf. With more than 90% of environmental PAHs being stored and transferred from soil,^90^ there is the potential for a natural bare soil pitch in a highly polluted area to pose a greater cancer risk than an artificial turf in the same area. Nevertheless, it is unclear what effect natural turf has on mitigating the transfer of COCs within soil, as opposed to the assumption of a bare soil pitch used by Peterson et al.^68^

Cancer risks below the EPA *de minimis* level, but above zero, were reported for inhalation exposure to benzo[a]pyrene,^47^ overall exposure to COCs,^68^ dermal exposure from rainfall runoff,^48,65^ direct dermal exposure,^48^ oral and dermal exposure to PAHs,^77^ dermal exposure to heavy metals^76^ and exposure to lead.^51^ Despite the use of similar models, many studies’ findings directly contrasted with each other.

A similar story regarding mixed HHRA outcomes was evident regarding non-carcinogenic risk. HQ scores of 1 or higher were reported for ingestion of cobalt in a child spectator^68^ and ingestion of arsenic, cobalt, thallium and zinc.^76^

HQ scores lower than 1 were reported for metal exposure via dermal contact;^67^ exposure to zinc, lead, copper and manganese via contact with rainfall runoff;^58^ exposure to COCs via direct dermal contact, dermal contact with rainfall runoff and inhalation;^48^ oral and dermal exposure to BPA and 6PPD;^77^ benzothiazole exposure via inhalation;^70^ exposure to benzothiazoles, phthalates and BPA;^51^ and overall exposure to COCs.^53,74^ Kim et al^60^ used a different scale, with HQs exceeding 0.1 representing a potential health risk, as their sole focus was on elementary school-age children. This 0.1 limit was exceeded for artificial turf with infill particles smaller than 250 µm but not for pitches with larger particle sizes.

### Limitations of the evidence used in the review

Artificial turf represents a large family of products produced by a range of manufacturers, each with varying compositions of infill and turf fibres, with varying densities of fibres and quantities of infill. Whilst some of the reported studies made efforts to individually report different compositions of infill when reporting total concentrations, many studies did not specify the infill type, simply referring to the infill as ‘crumb rubber’. Furthermore, some studies did not perform separate analyses on infill and turf fibres, instead analysing whole samples of artificial turf. These inconsistencies limit the ability to pinpoint which types of infill contain the highest concentrations of COCs or whether infill poses more of a potential hazard to health than turf fibres do. Such insights are needed with the upcoming EU ban on traditional crumb rubber infill, as whilst the artificial turf industry may look towards alternative infill types, the polyethene turf fibres used will likely remain unchanged.

Without long-term data on the health effects of artificial turf on users, HHRAs were the best available alternative until such data were compiled. Despite this, the quality of HHRAs is highly dependent on the strength of the researchers’ methodology and judgement, particularly in relation to the choice of input data used. The lack of standardised methodology for HHRAs determining the health risks of artificial turf also emphasises the need to scrutinise the input data used and the assumptions made in HHRAs. This scrutiny was challenging due to the incomplete reporting of methodologies; Zhang et al,^58^ Zhang et al,^65^ and Kim et al^64^ did not report the reference doses used for COCs, with Zhang et al^58^ and Zhang et al^65^ also not reporting the exposure subjects used. Pronk et al^51^ did not provide references for the reference doses used. As Zhang et al^58^ did not report the slope factors used, it is possible that they used the slope factors for chromium (VI) instead of total chromium (which was sampled), resulting in the authors overestimating the risk of dermal chromium exposure. In addition, the US EPA Integrated Risk Information System (IRIS) database used by the majority of HHRAs reviewed does not report dermal slope factors or reference doses, requiring these inputs to be calculated using the equivalent data for oral ingestion. Estimates for dermal doses are based on the actual dose absorbed, whereas oral dose-response relationships are based on the potential dose received.^92^ Without the use of pharmacokinetic modelling, such as via a bioaccessibility assay using synthetic sweat biofluid, uncertainty is placed on the dermal exposure risks calculated by some HHRAs. A challenge faced by performing HHRAs is achieving a balance between producing a conservative estimate of risk and ensuring that the risk calculated is reflective of real-world exposure scenarios. Conservative assumptions such as 100% bioavailability of a substance may also be assumed when bioavailability assays are not performed. This limits the validity of the outcomes provided by such HHRAs, as the assumptions made no longer reflect real-world exposure conditions. Pavilonis et al^67^ reported that at 100% lead bioavailability, there was a 34% probability of a child (2-7 years old) having a blood lead level over 5 µg/dl, whereas using the EPA recommended bioavailability values showed that there was a less than 0.5% probability of the child’s blood level exceeding the limit. Even in studies that performed total concentration analyses, studies such as Schneider et al^77^ chose to use conservative total concentration assumptions instead of what was measured, raising further questions on real-world applicability. When the concentration values used in HHRAs are far above real-world levels, the validity of statements claiming that particular COCs were ‘the biggest contributors to risk’ is questionable. An important factor to ensuring real-world applicability is the duration and frequency of exposure of users to the hazard investigated. Mohamed et al^74^ did not describe the activity of their selected user age groups, adding uncertainty to their findings. Only Kim et al^60^ and Kim et al^64^ performed surveys to determine locally representative average durations and frequencies of exposure to each group of artificial turf users, with Kim et al^64^ filming video footage to analyse user activity in greater detail. Acute exposure to COCs in children may also have a greater negative impact on health than chronic exposure in adults. For example, lead is a developmental neurotoxin, with children having up to eight times greater bioaccessibility of lead in the gastrointestinal tract than adults.^93^ Ruffino et al^48^ reported a simplistic exposition rate based on years of exposure, calculating a greater cancer risk in an adult player than in a child player, despite evidence from other studies suggesting the opposite.

Another important consideration is the synergistic toxicity that can occur from simultaneous exposure to multiple COCs. In terms of metal exposure, elements with a low covalent index have been shown to exhibit a neutralising effect on the toxicity of lower covalent index elements, whereas elements with a high covalent index exhibit a synergistic effect on toxicity.^94^ In vivo, heavy metals have also been shown to induce varying stress patterns in different organs, with stress induced in the gut by mercury, in the pharynx by cadmium and in the hypodermis by zinc.^95^ For other rubber additives, DEHP and DINP have been shown to exert synergistic effects on endocrine disruption,^96^ whilst 4-tert-octylphenol and BPA can exert synergistic effects on gestational stage malformation.^97^ Evidently, complex interactions to strengthen or weaken the overall toxicity between COCs may occur, which cannot be accounted for by the current exposure models. Many of the reviewed HHRAs calculated cumulative risk values which assume that the risks were additive across all COCs, as highlighted by Ginsberg et al,^70^ which does not reflect these complex interactions between COCs. The development of in vitro organotypic human models, combined with the power of systems biology, can enable future HHRA models to consider synergistic toxicity while simultaneously representing a more accurate predictor of the health impact posed by COCs.

### Limitations of the review process

We have made efforts to minimise selection bias by ensuring that a wide search for relevant studies was performed in this review by using multiple databases, maintaining a rolling search over several months, and not limiting the type of study included. We did not exclude non-English results from our search to mitigate language bias. Due to the heterogeneity of the included studies, we were not able to identify a preexisting risk of bias tool to evaluate all the studies appropriately. The risk of using a custom risk of bias tool that has not been evaluated through peer review was that a higher degree of subjectivity was introduced due to reviewers’ own biases. Although the level of subjectivity in our assessment could be critiqued, there was a high level of agreement between reviewers when evaluating the presence of bias across all papers.

Studies that provide useful evidence for the health impact of crumb rubber may have been missed due to the scope of this review, for example, studies exploring the toxicological impacts of COCs. We have purposely limited the scope of this paper to cover the crumb rubber used in artificial turf; however, its use extends as an additive to road surfaces and mulch for landscaping. Furthermore, microplastics represent a diverse group of materials with varying origins and compositions. Although we were unable to draw general conclusions about the health impact of microplastics, it provides evidence on the health impact of artificial turf, a key source of microplastics. A narrower scope was also used in the analysis of data from the included studies. Focus was placed on reporting the total concentration of COCs within crumb rubber and turf fibre, alongside bioaccessibility and HHRA conclusions. The air concentration of COCs and leaching from artificial turf were discussed in relation to how HHRAs were reported; however, our analysis did not extend beyond this. The associations between the age of the turf, or the size of infill particles and increasing or decreasing toxicity levels were also beyond the scope of this review. Differences in the composition of crumb rubber used in artificial turf were reported where possible; however, detailed comparisons, that is, coated versus uncoated ELT or coloured versus black infill, were not undertaken. We recommend that researchers consider dedicated reviews focusing on these areas, as including such in-depth analyses in this review would have exceeded the parameters of our project’s resources. Another area of review where the scope could be considered too narrow was the selection of COCs investigated. It was difficult to form a definitive list of COCs that pose the greatest threat to human health, as the isolated toxicity of a COC does not determine its toxicity; exposure occurs via a variety of routes and conditions. By referring to previous risk assessments, we have made efforts to ensure that emphasis was placed on COCs of interest in the study of human toxicology. We also acknowledge that future studies may uncover certain COCs to be more significant in their potential toxicity and that we can only make assumptions based on existing evidence. We acknowledge that the standards chosen to evaluate the safety of total concentration levels of COCs within artificial turf were all EU-based, whereas much of the studies’ sampling was conducted outside the EU. A range of non-EU standards were explored; none were deemed suitable due to a lack of comprehensiveness, availability or applicability to the reported results.

In contrast, we purposely kept the scope of the review broad regarding its investigation of exposure routes to COCs from artificial turf. Nevertheless, focusing on a single route of exposure in greater detail may have enabled a greater depth of analysis and comparison to identify clearer patterns in how COCs from artificial turf may pose specific health risks to humans.

## Conclusions

In summary, the reported COCs exceeded their relevant limits across the total concentration analyses performed by the studies included in this review. Exceedances were found to be similar between ELT and non-ELT crumb rubber samples (eg, median TPE and ELT PAH concentrations reported by Ruffino et al^48^), with notable exceedances also reported in turf fibre samples (eg, lead concentrations reported by Pavilonis et al^67^). Despite this, some of the COCs that exceeded their limits were not found to be bioaccessible, and the standards from which the limits were derived only denote an increased potential of hazard to human health. Exposure to PAHs and heavy metals was associated with carcinogenic risks above the EPA upper acceptable threshold for carcinogenic risk and above a HQ value of 1 for non-carcinogenic risk but no such risk was reported for exposure to rubber additives. The findings of some reported studies directly contrasted with those of other reported studies; however, the heterogeneity of the methodologies used in the included studies limited the ability to make accurate comparisons. Many of the reviewed HHRAs placed an emphasis on presenting a worst-case scenario evaluation of health risks posed by artificial turf as opposed to scenarios representative of real-world exposures. The methodologies used by the reviewed HHRAs did not consider the potential synergistic toxicities between chemicals present in artificial turf which may have impacted health risk. The reported results in which the upper acceptable threshold was exceeded, or the HQ was above 1 should be viewed as concerning, however, due to the range of assumptions and surrogates used for missing data, these results were unable to provide definitive evidence that a human health risk is present from the use of artificial turf in typical real-world scenarios. It could be argued that efforts should be made to mitigate any added health risk posed by artificial turf, regardless of how low or high the level of risk reported is. Indeed, with an upcoming EU ban on artificial turf infill to tackle environmental, health and sustainability concerns, other countries facing the same risks may choose to implement similar bans. Whilst such bans address the risk posed by artificial turf infill, artificial turf fibres contain COCs at similar or higher concentrations than infill and are also a source of microplastics. Users and installers of artificial turf surfaces may consider strategies to mitigate potential health risks, or simply avoid the installation or usage of artificial turf where vulnerable populations, that is, young children, are involved.

## Supplemental Material

sj-docx-1-ehi-10.1177_11786302241306291 – Supplemental material for Exploring the Human Health Impact of Artificial Turf Worldwide: A Systematic Review Supplemental material, sj-docx-1-ehi-10.1177_11786302241306291 for Exploring the Human Health Impact of Artificial Turf Worldwide: A Systematic Review by Sebastian Ryan-Ndegwa, Reza Zamani and Tanimola Martins in Environmental Health Insights
