# Supplementary material for: Exploring the Human Health Impact of Artificial Turf Worldwide: A Systematic Review
Source: Environ Health Insights. 2024 Dec 17;18:11786302241306291. doi: 10.1177/11786302241306291 (PMC11653453; doi:10.1177/11786302241306291)
Supplement: sj-docx-1-ehi-10.1177_11786302241306291 – Supplemental material for Exploring the Human Health Impact of Artificial Turf Worldwide: A Systematic Review [file sj-docx-1-ehi-10.1177_11786302241306291.docx]

**Supplemental Table 1: Study characteristics.** COCs = chemicals of concern, EPDM = ethylene propylene diene monomer rubber, TPE = thermoplastic elastomer**,** CP = chlorinated paraffin, EPA = Environmental Protection Agency

| **Authors** | **Country of sampling** | **Sampling method** | **Type of analysis performed** |
| --- | --- | --- | --- |
| **Armada et al. [75]** | Worldwide | - 78 crumb rubber samples (71 outdoor / seven indoor) from football pitches - From 17 countries covering four continents (North and South America, Europe, Asia and Africa). - Between 2-100g of sample were collected from each pitch | - Total concentration of COCs |
| **Benoit and Demars [73]** | USA | - 15 unused crumb rubber samples, nine for residential use and six for commercial use | - Total concentration of COCs - Air concentration of COCs (headspace analysis) - Leachate concentration of COCs |
| **Bocca et al. [54]** | Italy | - 32 crumb rubber samples were collected from 32 different playgrounds - Each playground was split into 12 sectors, with 250g of sample being collected from the centre of each sector. 50g of this sample from each sector was homogenised to make one sample per playground. | - Total concentration of COCs - Leachate concentration of COCs |
| **Celeiro et al. (a) [56]** | Spain | - 28 crumb rubber samples collected from football pitches (13 samples) and playgrounds (10 outdoor, five indoor samples). - Between 2-100g of sample were collected from each surface | - Total concentration of COCs |
| **Celeiro et al. (b) [50]** | Spain | - Crumb rubber samples collected from 15 artificial turf football fields. 20ml of rainfall runoff was collected from two of the fields - Headspace analysis of a single sample was performed to determine air pollution at the fields | - Total concentrations of COC - Concentration of COCs in rainfall runoff - Air concentration of COCs (via headspace analysis) |
| **Ginsberg et al. [70]** | USA | - see Simcox et al. [71] | - Human health risk assessment |
| **Graça et al. [76]** | Worldwide (EU + Chile) | - 103 crumb rubber samples (95 outdoor fields and eight indoor fields) collected at artificial turf pitches in 13 countries. - 3-100g of sample were collected from each pitch via spade | - Total concentration of COCs - Human health risk assessment |
| **Grynkiewicz-Bylina et al. [52]** | Poland | - 84 samples of recycled crumb rubber (Collected from 17 sports fields, 67 supplied by recycling companies) - Field sampling was conducted at 6 points across each field, then homogenised | - Total concentration of COCs - Leachate concentration of COCs |
| **Kawakami et al. [62]** | Japan | - 46 unused crumb rubber samples from 10 artificial turf field contractors (made from recycled tyres, industrial rubber, a combination or TPE) - Simulated human sweat, saliva, gastric and intestinal juices were used to determine bioaccessibilty | - Total concentrations of COCS - bioaccessibility of COCs |
| **Kim et al. (a) [60]** | South Korea | - Single sample of EPDM rubber powder grinded and analysed. Ingestion exposure characteristics of lead from sample determined via total content test, acid extraction, artificial digestion to determine bioavailability (synthetic gastric juice and synthetic duodenal juice) | - Total concentration of COCs - Bioaccessibility of COCs - Human health risk assessment |
| **Kim et al. (b) [64]** | South Korea | - Samples collected from 50 schools and were analysed in accordance with US EPA standards (US EPA 3062, US EPA 8100, US EPA 8061A) | - Total concentration of COCs - Bioaccessibility of COCs |
| **Kubota et al. [63]** | Japan | - 46 unused crumb rubber samples from 10 artificial turf field contractors (made from recycled tyres, industrial rubber, a combination, TPE or EPDM) - Simulated human sweat, saliva, gastric and intestinal juices were used to determine bioaccessibilty | - Total concentrations of COCS - Bioaccessibility of COCs |
| **Li et al. [72]** | USA | - 15 unused crumb rubber samples, five unused samples from five schools, eight unused recycled tyre samples, three unused alternative rubber materials and two samples collected from school fields | - Total concentration of COCs |
| **Marsili et al. [53]** | Italy | - Crumb rubber analysed from nine artificial turf football pitches (five samples of crumb rubber had not yet been applied on the field) | - Total concentration of COCs - Air concentration of COCs - Human health risk assessment |
| **Menichini et al. [47]** | Italy | - Athlete-worn air monitors used for sampling at two pitches (over two days) - Three static high-volume air samplers at two pitches (on-pitch / off-pitch / control) (over three days) - Crumb rubber samples from 13 pitches (600g from 12 sites in each pitch) | - Total concentration of COCs - Air concentration of COCs - Human health risk assessment |
| **Mohammed et al. [74]** | Egypt | - 15 crumb rubber samples from three groups of fields: nine samples from three football pitches in Giza, Egypt and six samples of virgin crumb rubber, three of which being exposed to sunlight, three of which without. | - Total concentration of COCs - Air concentration of COCs - Human health risk assessment |
| **Moreno et al. [57]** | Spain | - Analysis of ten samples of EPDM infill, three black crumb rubber samples and three coloured crumb rubber samples, all virgin. | - Total concentration of COCs |
| **Negev et al. [59]** | Israel | - Eight samples of new, uninstalled artificial turf | - Total concentration of COCs |
| **Nishi et al. [61]** | Japan | - 46 unused crumb rubber samples from 10 artificial turf field contractors (made from recycled tyres, industrial rubber, a combination or TPE) - Simulated human sweat, saliva, gastric and intestinal juices were used to determine bioaccessibilty | - Total concentrations of COCS - Bioaccessibility of COCs |
| **Pavilonis et al. [67]** | USA | - Newly manufactured crumb rubber infill (nine samples) - Turf fibre products (eight samples and seven samples from outdoor athletic fields - Bioaccessibility assessed using biofluids. Digestion: artificial saliva / gastric fluid / intestinal fluid. Dermal: sweat solution. Respiratory: synthetic lung solution | - Total concentration of COCs - Bioaccessibility of COCs - Human health risk assessment |
| **Peterson et al. [68]** | USA | - 76 outdoor air (from seven studies) - 17 indoor air samples (from two studies) | - Human health risk assessment |
| **Pronk et al. [51]** | Netherlands | - Rubber infill samples from 100 outdoor artificial turf pitches (96 football pitches, two korfball pitches and two Cruyff courts). - Six positions per pitch were sampled. - The infill in nine pitches which were found not to contain car tyre rubber were eliminated, leaving 546 total samples | - Total concentration of COCs - Leachate concentration of COCs - Air concentration of COCs - Human health risk assessment |
| **Ruffino et al. [48]** | Italy | - 1.2 kg of infill material collected at six outdoor artificial turf fields (from 12 positions in each field via extractor fan). - 1kg of natural soil collected from one field (control).  - Sample of gases and dust collected from field and near the field + centre of the city (control). | - Total concentration of COCs - Leachate concentration of COCs - Air concentration of COCs - Human health risk assessment |
| **Schillirò et al. [55]** | Italy | - Passive air sampling at five artificial turf football fields. Comparisons/controls were taken from a single clay football pitch and two urban meteorological stations - Sampling events took place over 24 hours, using high-volume air samplers placed at the top of the penalty area of the field - Sampling was performed over two periods: 14 days in June without prior playing activity (14 days prior) and over nine days in November during sports matches | - Air concentration of COCs - In-vitro mutagenicity assay |
| **Schneider et al. (3-parts) [49,77,78]** | Europe-wide | - 47 crumb rubber samples (40 outdoor / seven indoor) from artificial turf sports fields (football, rugby and school sports) - From 14 European countries (2.3 kg crumb rubber collected by Labosport staff from seven spots per field) - 29 samples were taken at recycling companies producing infill materials  - Simulated human sweat, saliva and gastric juice were used to determine bioaccessibilty | - Total concentration of COCs - Bioaccessibility of COCs - Air concentration of COCs - Human health risk assessment |
| **Simcox et al. [71]** | USA | - For total concentration analysis: 11 polyethylene-grass fields with crumb rubber infill (mixed indoor and outdoor) - For air concentration analysis: 5 polyethylene-grass fields with crumb rubber infill (four outdoor, one indoor) - Three to four volunteers performed soccer drills/scrimmage for two hours at each field (in addition to the control field) equipped with personal air monitors  - An on-pitch stationary air monitor was placed at a height of either three inches or six feet on field. - Upwind monitors were placed outside the pitch, 'usually on grass' - An additional 'community' monitor on a 'grass field near a busy road' was set up to control for background pollution. | - Total concentration of COCs - Air concentration of COCs |
| **Zhang et al. (a) [58]** | China | - Water run-off from three rainfall events at five athletic fields (only one field used artificial turf) - 300ml collected at beginning of rainfall and every 30mins until rain stops | - Concentration of COCs in rainfall runoff - Human health risk assessment |
| **Zhang et al. (b) [69]** | USA | - Seven crumb rubber samples (three from same field collected two months, three months and two years after installation) and one sample of artificial grass fibre. - Three samples were analysed using synthesised biofluids to assess bioavailability via ingestion (synthetic saliva, gastric fluid, intestinal fluid) | - Total concentration of COCs - Bioaccessibility of COCs |
| **Zhang et al. (c) [65]** | China | - Rainfall runoff analysed from drain outlet at a single artificial turf field. Sampling was conducted during three summer rainfall events, every 30 mins until rainfall ceased | - Concentration of COCs in rainfall runoff - Human health risk assessment |
| **Xie et al. [66]** | China | - Crumb rubber and grass fibre fragments analysed from three sports fields, including one playground | - Total concentration of COCs |
